# Clinical performance of E2Fs 1-3 in kidney clear cell renal cancer, evidence from bioinformatics analysis

**DOI:** 10.18632/genesandcancer.143

**Published:** 2017-05

**Authors:** Bin Liang, Jianying Zhao, Xuan Wang

**Affiliations:** ^1^ Department of bioinformatics, Key Laboratory of Cell Biology, Ministry of Public Health and Key Laboratory of Medical Cell Biology, Ministry of Education, College of Basic Medical Science, China Medical University, Shenyang, China; ^2^ Department of Clinical Laboratory, No.202 Hospital of PLA, Shenyang, China; ^3^ Graduate School, Jinzhou Medical University, Jinzhou, China; ^4^ Graduate School, Dalian Medical University, Dalian, China

**Keywords:** kidney cancer, prognosis, TCGA database, bioinformatics analysis

## Abstract

Extensive research on the E2F transcription factor family has led to numerous insights that E2Fs were involved not only in proliferation and tumorigenesis but also in apoptosis and differentiation. In the present study, we analyzed the differential expression of E2Fs1-3 genes, and also evaluated the impact of E2Fs 1-3 genes expression on clinical outcome from the Cancer Genome Atlas (TCGA) database. The results showed that E2F1, E2F2 and E2F3 expression was increased in KIRC tissues than matched normal tissues (E2F1, *P* < 0.001; E2F2, *P* < 0.001, E2F3, *P* = 0.001), respectively. E2F1, E2F2 and E2F3 were significantly different in metastasis status, lymph node status, stage, and T stage in KIRC patients (all *P* < 0.01). E2F1 and E2F2 had the sensitivity of 96.1% and 93.1% and the specificity of 87.2% and 91.7% in discriminating KIRC from normal controls. High E2F1, E2F2 and E2F3 expression were correlated to worsen overall survival (all *P* < 0.01), and high E2F3 expression had worse disease free survival (*P* = 0.0404). Multivariate Cox regression analysis revealed that E2F1 and E2F3 were independent prognostic factors for overall survival. Taken together, E2F1 and E2F2 may serve as valuable diagnostic markers for KIRC. Moreover, E2F1, E2F2 and E2F3 could provide valuable prognostic information for KIRC patients.

## INTRODUCTION

Kidney cancer, one of the considerable public health problems in the worldwide, is among the top ten most common malignancies in both men and women [1]. It is estimated that over 65,000 Americans are diagnosed with kidney cancer each year and nearly 13,000 die of this disease [1, 2]. Among them, clear cell renal cell carcinoma (ccRCC) is the most common histological subtype and accounts for 70%-80% of renal cancer cases [3]. Despite extensive efforts have been made to incorporate diverse molecular information for early diagnosis, better prognosis and treatment plans in the last decade, early stage ccRCC has an overall survival of 60-70%, and late stage ccRCC has a poor prognosis with 5-year survival of less than 10% [4]. ccRCC pathogenesis is a complex, multistage, and heritage-related process, and tumor genes are in the heterogeneous network of stromal, endothelial, innate inflammatory cells and specific immune cells surround or lay within the malignant tumor nests. Therefore, the identification of molecular markers that are predictive of ccRCC aggressiveness and patient outcome has the potential to improve the ability to manage patients and new molecular drug targets.

The E2F family of transcription factors consists of eight proteins (E2F1, E2F2, E2F3, E2F4, E2F5, E2F6, E2F7 and E2F8) that bind to the consensus E2F motif (TTTCGCGC) [5]. Mounting evidence has identified that E2F family members involved in DNA synthesis, cell cycle, cell differentiation, and apoptosis [6-9]. The E2Fs members are divided into two subfamilies: E2Fs 1-3 are activators of transcription, whereas E2Fs 4-8 act as repressors [10]. There is growing evidence that deregulation of the E2F family itself is crucially involved in carcinogenesis [11]. However, most of the studies done thus far focused on the deregulation of proliferation-promoting members of the E2F family, especially E2F1, E2F2, and E2F3. E2F1 is the first cloned member and plays an imperative role in cell fate control. Ma X, *et al*. reported that E2F1 over-expression contributed significantly to kidney cancer cell proliferation, migration and invasion *in vitro* [12]. In addition, miR-155 functions as a tumor-promoting microRNA by targeting E2F2 in ccRCC [13]. Recent study reported that E2F3 acted to transactivate HIF-2α transcription in ccRCC, which in turn exerted a serial effect on the pivotal epithelial-mesenchymal transition-related genes [14].

Although numerous studies have reported that E2Fs 1-3 expression was of clinical significance in different cancers, little is known about the relationship between E2Fs 1-3 expression and prognosis in ccRCC. In the present study, we analyzed the Cancer Genome Atlas (TCGA) database to evaluate the differential expression of E2Fs1-3 genes, and also evaluated the impact of E2Fs 1-3 genes expression on clinical outcome. Consequently, this study enhanced the understanding of E2Fs 1-3 prognostic roles in ccRCC, and also provided a feasible approach with bioinformatics guidance in complex diseases.

## RESULTS

### Patient characteristics from TCGA database

The information of all patients downloaded from TCGA Kidney Renal Clear Cell Carcinoma (KIRC) database was list in Table 1. The patients included 344 males and 186 females. The median age at diagnosis was 60 years (range, 26 - 90 years). All of the patients were assessed according to the system for staging primary tumor/regional lymph node/distance metastasis (TNM) described in the AJCC cancer staging manual. The median of overall survival (OS) was 39.32 months (range, 0-149.05 months) and the median of disease free survival (DFS) was 36.37 months (range, 0-133.84 months).

**Table 1:** Characteristics of KIRC patients in TCGA database

Variables	Case, *n* (%)
Age at diagnosis	
≤60	265 (50.0%)
>60	265 (50.0%)
Sex	
female	186 (35.1%)
male	344 (64.9%)
Tumor size	
≤2cm	398 (75.1%)
>2cm	97 (18.3%)
NA	35 (6.6%)
Metastasis	
M0	418 (78.9%)
M1	78 (14.7%)
MX	31 (5.8%)
NA	3 (0.6%)
Lymph node status	
N0	238 (44.9%)
N1-2	16 (3.0%)
NX	276 (52.1%)
Stage	
I+II	322 (60.8%)
III+IV	204 (38.5%)
NA	4 (0.7%)
T stage	
T1+T2	340 (64.2%)
T3+T4	189 (35.7%)
NA	1 (0.1%)

NA, none available.

### E2F1, E2F2 and E2F3 levels in KIRC patients and normal controls

**Figure 1:**
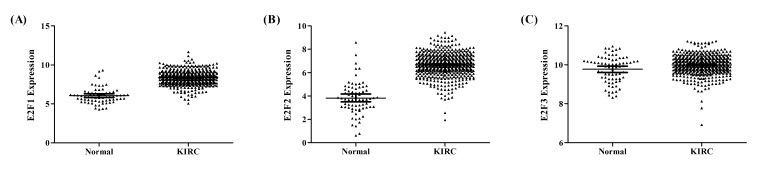
E2F1, E2F2 and E2F3 levels in KIRC patients and matched normal controls **A.** E2F1 (*P* < 0.001); **B**. E2F2 (*P* < 0.001); **C**. E2F3 (*P* = 0.001).

As shown in Figure 1, E2F1, E2F2 and E2F3 mRNA levels were increased in KIRC tissues than matched normal tissues (E2F1, *P* < 0.001; E2F2, *P* < 0.001, E2F3, *P* = 0.001), respectively. Moreover, E2F1, E2F2 and E2F3 were on average 1.38-fold, 1.74-fold, and 1.02-fold over-expressed in KIRC tissues.

### Association between E2F1, E2F2, and E2F3 levels and the clinical characteristics in KIRC patients

We explored the relationship between E2F1, E2F2, and E2F3 expression and clinical features in KIRC patients. We found E2F1 were significantly different in metastasis status (*P* = 0.015), lymph node status (*P* < 0.001), stage (*P* = 0.001), and T stage (*P* < 0.001) (Table 2). E2F2 expression was found to be significantly different in metastasis status, lymph node status, stage, and T stage (all *P* < 0.001) (Table 3). E2F3 expression was also significantly different in tumor size (*P* = 0.049), metastasis status (*P* = 0.016), lymph node status (*P* = 0.039), stage (*P* < 0.001), and T stage (*P* < 0.001) (Table 4). However, no significant difference was observed in age, gender, and tumor size for E2F1 and E2F2, and no significant difference in age and gender for E2F3 expression (all *P* > 0.05).

**Table 2:** Association between E2F1 expression and clinical characteristics in KIRC patients

Variables	Numbers	E2F1 expression	*P* value
Age at diagnosis			
≤60	265	4.37 ± 2.73	0.733
>60	265	4.28 ± 3.33	
Sex			
female	186	4.10 ± 2.64	0.217
male	344	4.44 ± 3.23	
Tumor size			
≤2cm	398	4.32 ± 3.04	0.765
>2cm	97	4.22 ± 2.86	
Metastasis			
M0	418	4.16 ± 3.03	0.015
M1	78	5.06 ± 2.75	
Lymph node status			
N0	238	4.19 ± 2.75	<0.001
N1-2	16	7.12 ± 2.89	
Stage			
I+II	322	3.96 ± 2.35	0.001
III+IV	204	4.86 ± 3.82	
T stage			
T1+T2	340	3.96 ± 2.37	<0.001
T3+T4	189	4.99 ± 3.90	

**Table 3:** Association between E2F2 expression and clinical characteristics in KIRC patients

Variables	Numbers	E2F2 expression	*P* value
Age at diagnosis			
≤60	265	4.90 ± 3.36	0.668
>60	265	4.77 ± 3.53	
Sex			
female	186	4.66 ± 3.30	0.389
male	344	4.93 ± 3.52	
Tumor size			
≤2cm	398	4.81 ± 3.39	0.299
>2cm	97	5.21 ± 3.55	
Metastasis			
M0	418	4.59 ± 3.23	<0.001
M1	78	6.35 ± 3.74	
Lymph node status			
N0	238	4.83 ± 3.23	<0.001
N1-2	16	9.33 ± 5.47	
Stage			
I+II	322	4.25 ± 2.99	<0.001
III+IV	204	5.72 ± 3.90	
T stage			
T1+T2	340	4.28 ± 3.00	<0.001
T3+T4	189	5.83 ± 3.94	

**Table 4:** Association between E2F3 expression and clinical characteristics in KIRC patients

Variables	Numbers	E2F3 expression	*P* value
Age at diagnosis			
≤60	265	1.11 ± 0.35	0.623
>60	265	1.13 ± 0.36	
Sex			
female	186	1.13 ± 0.36	0.753
male	344	1.12 ± 0.36	
Tumor size			
≤2cm	398	1.11 ± 0.36	0.049
>2cm	97	1.19 ± 0.32	
Metastasis			
M0	418	1.11 ± 0.34	0.016
M1	78	1.21 ± 0.40	
Lymph node status			
N0	238	1.15 ± 0.35	0.039
N1-2	16	1.35 ± 0.52	
Stage			
I+II	322	1.07 ± 0.33	<0.001
III+IV	204	1.21 ± 0.38	
T stage			
T1+T2	340	1.07 ± 0.34	<0.001
T3+T4	189	1.22 ± 0.38	

**Table 5:** Univariate and multivariate Cox regression analysis in KIRC patients

	Univariate analysis		Multivariate analysis	
	HR (95% CI)	*P* value	HR (95% CI)	*P* value
Overall survival (OS)				
Age (≥ 60 *vs*. <60)	1.753 (1.290-2.383)	<0.001	1.641 (1.194-2.255)	0.002
Gender (male *vs*. female)	0.953 (0.699-1.299)	0.758		
Size (>2cm *vs*. ≤2cm)	1.214 (0.859-1.718)	0.272		
Clinical stage (III+IV *vs*. I+II)	3.858 (2.807-5.301)	<0.001	3.260 (2.350-4.522)	<0.001
T stage (T3+T4 *vs*. T1+T2)	3.178 (2.347-4.305)	<0.001		
E2F1 level (high *vs*. low)	1.539 (1.137-2.083)	0.005	1.418 (1.035-1.942)	0.029
E2F2 level (high *vs*. low)	1.537 (1.133-2.086)	0.006		
E2F3 level (high *vs*. low)	1.813 (1.329-2.474)	<0.001	1.490 (1.080-2.055)	0.015
Disease-free survival (DFS)				
Age (≥ 60 *vs*. <60)	1.352 (0.950-1.923)	0.094		
Gender (male *vs*. female)	1.491 (1.000-2.222)	0.051		
Size (>2cm *vs*. ≤2cm)	1.459 (0.973-2.188)	0.068		
Clinical stage (III+IV *vs*. I+II)	6.262 (4.231-9.269)	<0.001	6.487 (4.323-9.734)	<0.001
T stage (T3+T4 *vs*. T1+T2)	4.392 (3.057-6.311)	<0.001		
E2F1 level (high *vs*. low)	1.324 (0.929-1.887)	0.120		
E2F2 level (high *vs*. low)	1.389 (0.973-1.983)	0.071		
E2F3 level (high *vs*. low)	1.501 (1.046-2.154)	0.027		

### Diagnostic performances of E2F1, E2F2, and E2F3 in KIRC patients

**Figure 2:**
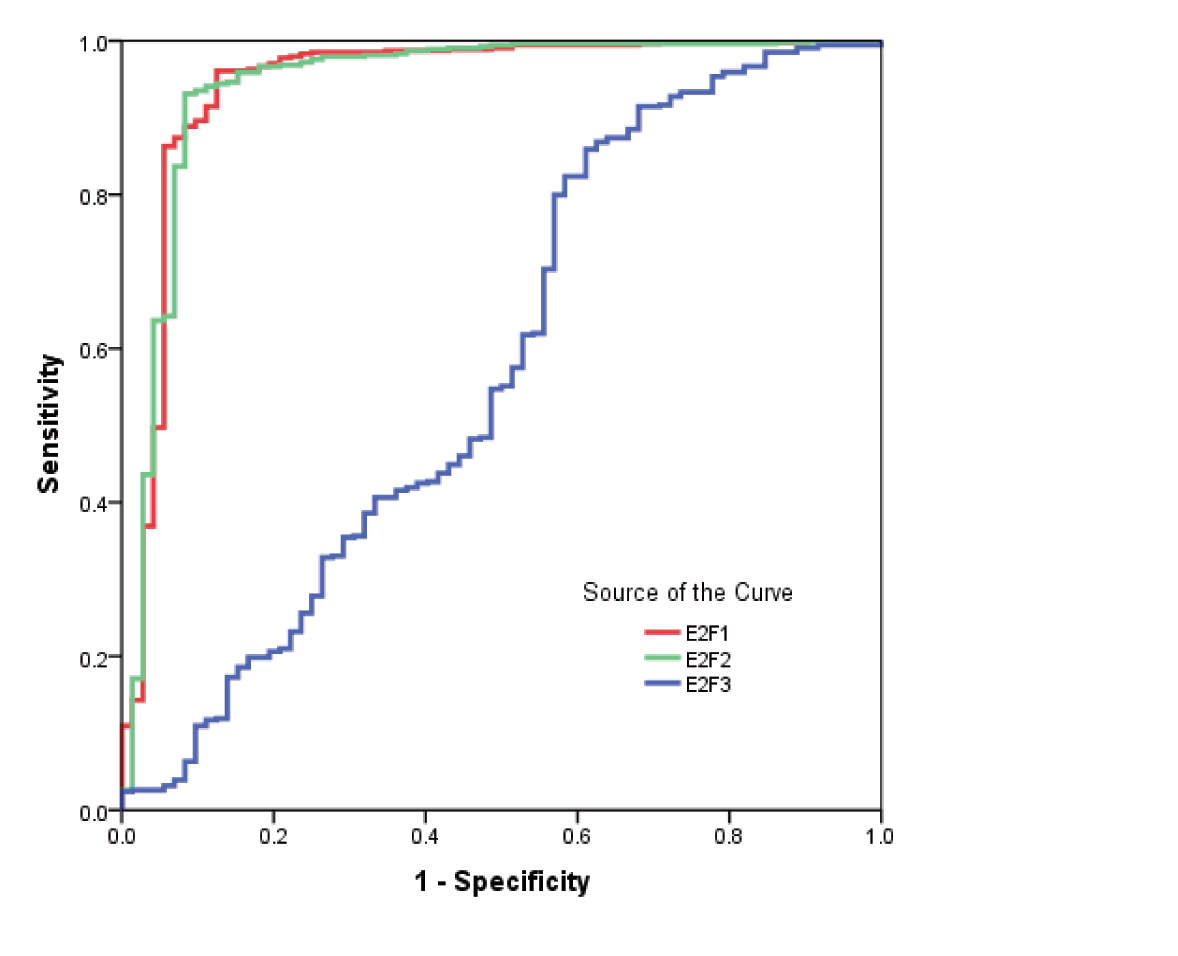
ROC curves for E2F1, E2F2 and E2F3 in discriminating KIRC from normal controls

The diagnostic performances of E2F1, E2F2 and E2F3 were examined by performing receiver operating characteristic (ROC) curve analysis. As shown in Figure 2, the area under curve (AUC) values of E2F1and E2F2 were 0.944 (95%CI: 0.904-0.983) and 0.942 (95%CI: 0.903-0.982), respectively. The sensitivity and specificity reached 96.1% and 87.2% for E2F1, and 93.1% and 91.7% for E2F2 in discriminating KIRC from normal controls. But, the AUC of E2F3 was 0.579 (95%CI: 0.498-0.660), providing a sensitivity of 54.7% and a specificity of 51.4%.

### Prognostic performances of E2F1, E2F2, and E2F3 in KIRC patients

**Figure 3:**
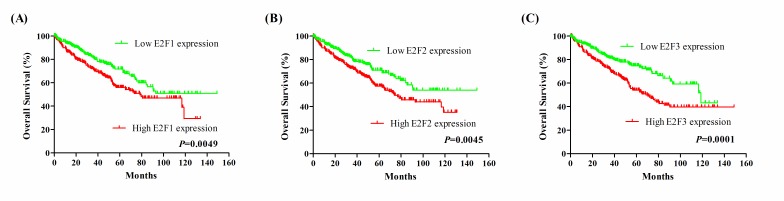
Kaplan-Meier survival curves for OS in KIRC patients stratified by median of E2F1, E2F2 and E2F3 **A.** E2F1; **B.** E2F2; **C.** E2F3.

**Figure 4:**
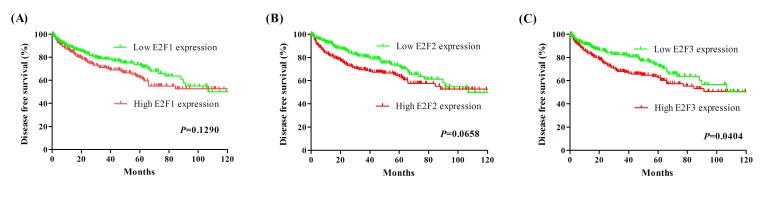
Kaplan-Meier survival curves for DFS in KIRC patients stratified by median of E2F1, E2F2 and E2F3 **A.** E2F1; **B.** E2F2; **C.** E2F3.

Based on the median of E2F1, E2F2 and E2F3, we performed the Kaplan-Meier analysis to estimate patient's OS and DFS. As shown in Figure 3, Kaplan-Meier survival curve showed that patients with high E2F1 expression had worse OS in KIRC patients (hazard ratio [HR] = 1.537 [95%CI: 1.139-2.069], *P* = 0.0049). High E2F2 expression was correlated with worse OS in KIRC patients (HR = 1.550 [95%CI: 1.143-2.075], *P* = 0.0045). Furthermore, high E2F3 expression was also found to be correlated with worse OS in KIRC patients (HR = 1.813 [95%CI: 1.325-2.405], *P* = 0.0001). Then, Figure 4 showed that the DFS rate in high E2F3 expression group was significantly lower than the low E2F3 expression group (HR = 1.445 [95%CI: 1.018-2.060], *P* = 0.0404). But, high E2F1 and E2F2 expression was found no correlation with DFS in KIRC patients (E2F1: HR = 1.311 [95%CI: 0.924-1.860], *P* = 0.1290; E2F2: HR = 1.392 [95%CI: 0.979-1.982], *P* = 0.0658).

To assess whether E2F1, E2F2, and E2F3 were the independent prognostic factors for KIRC patients, we conducted a univariate and multivariate Cox regression analysis, including age, gender, stage, T stage, and E2F1-3 expression levels. In the univariate Cox regression analysis, higher E2F1 (HR = 1.539 [95%CI: 1.137-2.083], *P* = 0.005), E2F2 (HR = 1.537 [95%CI: 1.133-2.086], *P* = 0.006), and E2F3 (HR = 1.813 [95%CI: 1.329-2.474], *P* < 0.001) expression was correlated with shorter OS in KIRC patients. Also, age (HR = 1.753 [95% CI: 1.290-2.383], *P* < 0.001), stage (HR = 3.858 [95% CI: 2.807-5.301], *P* < 0.001), and T stage (HR = 3.178 [95% CI: 2.347-4.305], *P* < 0.001) were correlated with OS in KIRC patients. Multivariate COX regression analysis revealed that age (HR = 1.641 [95%CI: 1.194-2.255], *P* = 0.002), stage (HR = 3.260 [95%CI: 2.350-4.522], *P* < 0.001), E2F1 (HR = 1.418 [95%CI: 1.035-1.942], *P* = 0.029) and E2F3 (HR = 1.490 [95%CI: 1.080-2.055], *P* = 0.015) were independent prognostic factors for OS. Subsequent univariate and multivariate Cox regression models were conducted to determine the independence of the prognostic power of E2F1-3 in KIRC patients’ DFS. In the univariate Cox regression analysis, clinical stage (HR = 6.262[95% CI : 4.231-9.269], *P* < 0.001), T stage (HR = 4.392 [95% CI: 3.057-6.311], *P* < 0.001), E2F3 expression (HR = 1.501 [95% CI: 1.046-2.154], *P* = 0.027) were correlated with DFS in KIRC patients. However, multivariate Cox regression revealed that only advanced clinical stage (HR = 6.487 [95% CI: 4.323 - 9.734], *P* < 0.001) could predict a worse prognosis on DFS for KIRC patients.

## DISCUSSION

Renal cell carcinoma represents 3 to 5 % of adult solid malignant tumors and is the third most frequent urological malignancy. It is estimated that median 5-year survival rates are 95 % for stage I, 88 % for stage II, 59 % for stage III, and only 20 % for stage IV [15, 16]. Therefore, it is requisite to investigate the molecular mechanism of renal cell carcinoma, formulate rational treatment, and provide novel therapeutic targets. To date, the roles of E2F activators in carcinogenesis and prognosis in many cancers have been confirmed, but, the method of further bioinformatics analysis has never been reported. In the present study, our findings provide evidence that the E2Fs 1-3 expression levels in KIRC patients were higher than matched normal controls. We explored the relationship between E2Fs 1-3 and the clinical characteristics as well as the diagnostic value of E2Fs 1-3 in KIRC patients. Moreover, univariate and multivariate Cox regression analysis demonstrated that E2F1 and E2F3 were independent prognostic factors for overall survival.

Over the past decades, extensive research on the E2F transcription factor family has led to numerous insights that E2Fs were involved not only in proliferation and tumorigenesis but also in apoptosis and differentiation [17, 18]. E2F1, the most thoroughly learned member of the E2F activator, can trigger diverse aberrant transcription processes that may dominate malignancy. Mounting evidence indicated that E2F1was a key regulator of the G1/S transition by inducing cell cycle protein including CDC2, CDC25a, and cyclin E [19]. Recent studies have shown that E2F1 can promote cell invasion and chemoresistance, though the targets underlying these processes are still poorly defined [20]. Moreover, high levels of E2F1 were correlated closely with ccRCC development and metastasis, and could augment EMT-related induction [21]. E2F2, located on 1p36, regulates lots of cell progresses such as cell cycle, proliferation and tumorigenesis [22]. Yuwanita I, *et al*. reported that *E2F2* loss results in increased metastasis in breast cancer, potentially functioning through a *PTPRD* dependent mechanism [23]. Interestingly, Li Chen, *et al*. reported that high E2F2 expression was associated with increasing tumor size and advanced clinical stage which indicated that E2F2 expression might be served as a promising hallmark of lung cancer outcomes [24]. Li T, *et al*. showed that E2F2 acted as a tumor suppressor in colon cancer by repressing the expression of survivin and regulating the expression of CCNA2, C-MYC, MCM4 and CDK2 [25]. Therefore, E2F2 may act as either a tumor suppressor or an activator in different cancer type. E2F3, encoding two different proteins, E2F3a and E2F3b, has been suggested to play a role in transcription activation. Unlike E2F1, E2F3 appears to be important for the efficient induction of the S phase in cycling cells [26]. There is substantial evidence supporting the importance of E2F3 in controlling cell cycle progression and proliferation in neoplastic and non-neoplastic cells [27]. Previous publications reported that E2F3 was amplified or over-expressed in several tumors, including bladder [28], prostate [29], kidney [14], and lung cancer [30]. Qiu M, *et al*. suggested that microRNA-429 (miR-429), a modulator of epithelial-to-mesenchymal transition, plays a crucial role in tumorigenesis and tumor progression by direct targeting of E2F3 in renal cell carcinoma [31]. We downloaded microRNA sequencing data from TCGA database and found that miRNA-429 was down-regulated in KIRC tissues compared with matched normal tissues (Fold change = -3.31 fold, *P* < 0.001, FDR < 0.001, data not shown). In the present study, we found E2F1 and E2F3 were up-regulated in KIRC patients, which is in similar to Ma X, *et al*. [12] and Gao Y, *et al*. [14] studies. Additionally, Gao Y, *et al*. also demonstrated that E2F2 acts as a tumor suppressor in renal clear cell cancer [13]. But, in our study, TCGA KIRC dataset revealed that E2F2 expression was significantly higher in KIRC patients than matched normal controls. Therefore, more research is needed to better understand the roles of E2Fs 1-3 in KIRC patients. Here, to gain insight into the function of E2Fs 1-3, we analyzed the relationship between E2Fs 1-3 expression and clinical features, such as age, gender, tumor size, metastasis, lymph node status, and TNM stage. The results suggested that E2F1, E2F2 and E2F3 were significantly associated with metastasis status, lymph node status, stage, and T stage, indicating E2F1-3 play important roles in the progression of KIRC.

The diagnostic values of E2F1, E2F2 and E2F3 in the detection of KIRC were evaluated using ROC curves. E2F1 and E2F2 had the sensitivity of 96.1% and 93.1%, the specificity of 87.2% and 91.7%, and the AUC of 0.944 and 0.942, suggesting that measuring E2F1 and E2F2 levels are the promising biomarkers for KIRC diagnosis. Moreover, we analyzed the association of E2Fs 1-3 with survival time according to TCGA dataset. The high E2F1, E2F2 and E2F3 expression was related to the reduction in OS, and high E2F3 expression was associated with decreased DFS, as shown by the Kaplan-Meier curves. However, multivariate Cox regression analysis revealed that E2F1 and E2F3 were the independent prognostic factors for patients’ overall survival.

## CONCLUSION

Altogether, the present study helped us to identify E2Fs 1-3 were involved in the progression of KIRC. Moreover, E2F1 and E2F2 had preferable diagnostic performance in discriminating KIRC from normal controls. Moreover, E2F1 and E2F3 were the independent prognostic factors for patients’ overall survival. However, the mechanisms of three genes impacting on the prognosis remain unclearly. Therefore, further studies are needed to verify our analysis and elucidate the molecular mechanisms, so as to provide a precise understanding of E2Fs 1-3 function in predicting the prognosis of KIRC.

## MATERIALS AND METHODS

### Patient and sample data extracted from TCGA database

The mRNA expression data of normal and tumor tissues were obtained through TCGA's online data portal site (https://cancergenome.nih.gov/). TCGA can be used to analyze complicated clinical profiles and cancer genomics. The recent publication of TCGA Kidney Renal Clear Cell Carcinoma (KIRC) project has provided an immense wealth and breadth of data, providing an invaluable tool for confirmation and expansion upon previous observation in a large data set containing multiple data types. The mRNA sequencing data (530 KIRC patients and 72 matched normal controls) were downloaded from the TCGA KIRC database. Clinical information for each patient included age, gender, tumor size, metastasis status, lymph node status, clinical stage, T stage, disease free survival, and overall survival.

### Analysis of E2F1, E2F2 and E2F3 expression in KIRC patients

The expression levels of E2F1, E2F2 and E2F3 were compared between KIRC and normal controls. Then, fold-changes (KIRC/normal) were used to measure the degrees of E2F1, E2F2, and E2F3 changes between KIRC tissues and matched normal controls. We further analyzed the association of E2F1, E2F2, and E2F3 with different clinical features, which include age, gender, tumor size, metastasis, lymph node status, clinical stage, and T stage.

### Diagnosis and prognosis analysis

The diagnostic performance of E2F1, E2F2 and E2F3 were evaluated using ROC curves. To judge the superiority or inferiority of three genes, the AUC was determined. For survival analysis, OS was assessed from the day of diagnosis to the day of last follow-up, while DFS was defined as the time from the day of the first complete remission to the day of first relapse or death. OS and DFS curves were established according to the Kaplan-Meier method and were compared using the log-rank test. In addition, univariate and multivariate Cox regression models were used to identify the prognostic effects of clinical features and E2Fs 1-3. A *P*-value of less than 0.05 was considered to be significant.

### Statistical analysis

SPSS 22.0 (SPSS, Inc., Chicago, IL, USA) was applied for the statistical analysis. The Mann-Whitney U test was used to compare the expression of the three genes in terms of different clinical variables (age, gender, tumor size, metastasis, lymph node status, clinical stage, and T stage). *P* < 0.05 was considered statistically significant (two-sides).

The study was supported and funded by the National Science Foundation of China (No. 81301835).
